# Key Information Extraction Algorithm of Different Types of Digital Archives for Cultural Operation and Management

**DOI:** 10.1155/2022/3459605

**Published:** 2022-08-29

**Authors:** Xiulun Ma

**Affiliations:** University of Jinan, Jinan 250022, China

## Abstract

In order to improve the effect of key information extraction from digital archives, a key information extraction algorithm for different types of digital archives is designed. Preprocess digital archive information, taking part of speech and marks as key information. Self-organizing feature mapping network is used to extract the key information features of digital archives, and the semantic similarity calculation results are obtained by combining the feature extraction results. Combine with mutual information collection, take that word with the highest mutual information value as the collection cent, traverse all keywords, and take the central word as the key information of digital archives to complete the extraction of key information. Experiments show that the recall rate of the algorithm ranges from 96% to 99%, the extraction accuracy of key information of digital archives is between 96 and 98%, and the average extraction time of key information of digital archives is 0.63 s. The practical application effect is good.

## 1. Introduction

Generally speaking, there are no management problems in the spontaneous stage of cultural production. Cultural production and business activities are the product of commodity economy [1, 2]. When material and cultural production develop to a certain extent, social division of labor is further clarified, and professionals and professional groups engaged in cultural production appear, both the ruling class and the ruled class try to use cultural production to serve the interests of their own class, which is the conscious stage of cultural production [3]. Only at the conscious stage of cultural production can cultural operation and management be put on the agenda. In a modern capitalist society, everything into a commodity culture products without exception has become a part of capitalists, for-profit special goods. The vast majority of cultural activities are restricted by the value of commodity production rules [4]. The tendency of commercialization of cultural production and cultural activities has become a common social phenomenon in the field of capitalist culture. The law of market economy dominates the management of cultural operations and management activities, and the quality of management is the key to the success or failure of specific cultural products in the free competition of the cultural market. Especially with the rapid development of social economy, different types of digital archives are gradually increasing, the main products of these are social culture, in order to better manage for these different types of digital files, need to study a new key to different types of digital archives information extraction algorithm, to enhance the management level of the digital scheme. Therefore, it is of great significance to study a key information extraction algorithm of different types of digital archives.

In the digital archives, information extraction is an important research topic, reference [5] proposed a digital book records mass data fast extraction algorithm. Based on the range characteristics of large-scale data attributes, the distribution samples of digital book archive data are divided into multiple subintervals to achieve data classification. By constructing a neuron model, the error terms of output are determined according to the data output of the hidden layer and output layer, and the weight of each layer of the BP neural network is adjusted. This method builds a fast extraction model based on BP neural network and realizes the fast extraction of massive archive data. Reference [6] proposed a key information extraction algorithm based on TextRank and cluster filtering. First, the key information is extracted and vectorized for Word2Vec. Then, TextRank is improved by constructing a graph model integrating word eigenvalues and edge weights, and the stable graphs obtained by iterative convergence are merged and clustered to form clusters. Then, a cluster quality evaluation formula was designed for cluster filtering, and TextRank was applied to form the final clustering. Finally, annotate the information type of the cluster. For testing the text, by comparing the key information vector distance cluster heart vector and the words information types, combines information type and key information to get the key of the text information. Reference [7] proposed a hidden Markov model based on an improved extraction algorithm of key information extraction. The web document is converted into D0M tree and preprocessed, and the information item to be extracted is mapped to state and the observation item to be extracted is mapped to vocabulary. The improved hidden Markov model is used to extract key information of the text. Reference [8] proposed a key information extraction algorithm based on word vector and location information. Vector representation model by word learning vector of each word in the target document said, will the reflect of the latent semantic relations between the word and the word vector combined with location feature fusion to the PageRank score model, choose a few top words or phrases as the key target document information, in order to complete the digital archives of key information extraction. Reference [9] proposed a key information extraction algorithm of unstructured text in the knowledge database. Six yuan group was used to optimize the hidden Markov model, probability model, and smooth processing of incomplete training samples. Initialization and termination operations were carried out for the sequences of observation values released at different times to obtain the optimal state sequence. After decoding the observation sequence, the positive sequence and reverse sequence were obtained by comparing them to filter out the states without decoding ambiguity and complete ambiguity elimination. According to the maximum probability state sequence, the text key information to be extracted is defined and the key information is extracted.

However, the above-mentioned key information extraction algorithm is suitable for different types of digital files, and the effect is not ideal because the boundary of key information extraction is uncertain. Therefore, this paper designs a new key information extraction algorithm for different types of digital archives. Firstly, the algorithm divides the main categories of key information, takes parts of speech and marks as features, and introduces the self-organizing feature mapping neural network to traverse the center of word set, thus realizing the extraction of key signals quickly and accurately. The effectiveness of the algorithm is verified by experiments.

## 2. Materials and Methods

### 2.1. Digital Archive Processing

#### 2.1.1. The Text Participle

In the process of cultural management, there are many types of digital archives. Before extracting the key information of different types of digital archives, it is necessary to preprocess the key information of digital archives. The preprocessing process includes word segmentation and marking. Word segmentation refers to classifying the words in the text and setting the marks according to the categories, which lays the foundation for the key information extraction of digital archives in the future [10].

**Figure 1:**
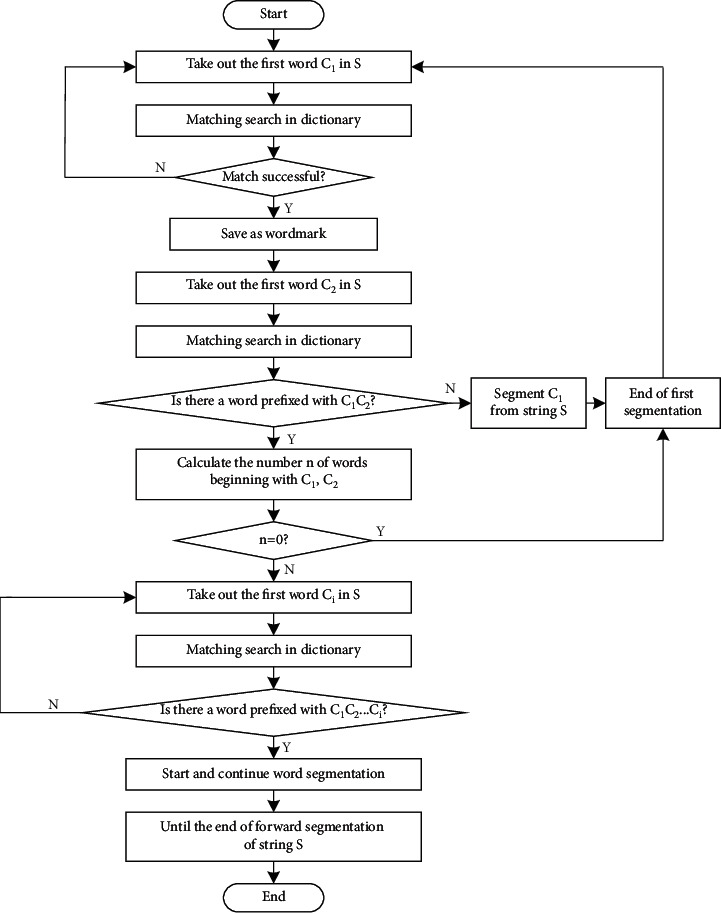
Text word segmentation and the process of removing stop words.

The difference between the word segmentation process of the reverse maximum matching algorithm and the forward maximum matching algorithm is that the scanning of the reverse maximum matching algorithm starts from the end of the string. Each unsuccessful match removes the preceding word until the match is successful. Then the basic idea of the bidirectional maximum matching algorithm is: When segmenting different types of digital archives information, firstly, a forward word-for-word maximum matching algorithm is applied to the character string to be processed, then a reverse word-for-word maximum matching algorithm is applied, and the output result is used to complete the word segmentation processing. Assuming bidirectional maximum matching word segmentation for *S*=(*C*_1_, *C*_2_,…, *C*_*i*_), the algorithm process can be described as follows:

1. First take out the first word *C*_1_ in *S*, and search in the dictionary to see if there are any words with *C*_1_ as the prefix. If there are, save them as word marks [11].
2. Take a word *C*_2_ from *S* and match it with the dictionary to determine whether there is a word with *C*_2_ as the prefix.
3. If it does not exist, split *C*_1_ from string *S*, ending with a word split.
4. If there is, to determine whether *C*_1_*C*_2_ into words, calculate the number *n* headed by *C*_1_*C*_2_ words.
5. If *n*=0, the participle ends once [12].
6. If *n* is not 0, then take a word *C*_*i*_ from *S* and match it with the dictionary to determine whether there is a word prefixed with *C*_1_, *C*_2_,…, *C*_*i*_.
7. If yes, go to Step 6.
8. If it does not exist, split *C*_1_, *C*_2_,…, *C*_*i*−1_ from string *S*, ending with a word split.
9. Continue word segmentation from string *C*_*i*_ of *S*, repeat the above steps until the end of string *S* forward segmentation.
10. Take out the last word *C*_*n*_ in *S* and match it in the dictionary to find whether there is a word with suffix *C*_1_. If so, save it as a word mark [13].
11. Then take out a word *C*_*n*−1_ from *S* and match it with the dictionary to judge whether there is a word with suffix *C*_1_*C*_2_.
12. If it does not exist, it splits *C*_*n*_ from string *S*, ending with a word split.
13. If there is, then judge whether *C*_*n*−1_*C*_*n*_ is a word and count the number of words starting with *C*_*n*−1_*C*_*n*_, expressed by *n*.
14. If *n*=0, then the participle ends.
15. If *n* is not 0, take out a word *C*_*i*_ from *S* and match it with the dictionary to determine whether there is a word with *C*_*i*_,…, *C*_*n*−1_*C*_*n*_ as the suffix.
16. If yes, go to Step (15).
17. If it does not exist, *C*_*i*_,…, *C*_*n*−1_*C*_*n*_ will be cut out from string *S* and a word segmentation will end.
18. Continue word segmentation from word *C*_*i*_ of string *S*, and repeat the above steps until the end of reverse segmentation of string *S*, so as to remove the stop word. The specific implementation process is shown in Figure 1.

#### 2.1.2. The Part of Speech Tagging

**Figure 2:**
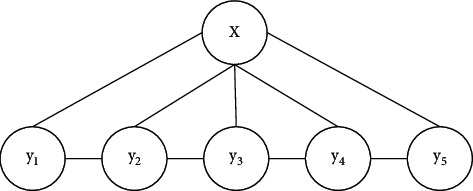
Chained conditional random fields.

Part of speech is a grammatical attribute of vocabulary, which generally indicates the type of a word in the corpus. Part-of-speech tagging refers to the process and method of tagging the part of speech of each word. Some words contain multiple parts of speech, with different parts of speech and completely different ways of expression [14, 15]. However, in general, when a word contains one or more parts of speech, the frequency of its commonly used parts of speech is far greater than that of other parts of speech, so the accuracy of POS tagging can be ensured on the whole, and the POS tagging method can be applied to most application scenarios [16]. Conditional Random Field Algorithm (CRF) was proposed by Lafferty et al. in 2001. It is an undirected graph model combining the characteristics of the maximum entropy model and hidden Markov model. In recent years, good results have been achieved in sequence tagging tasks such as word segmentation, part-of-speech tagging, and named entity recognition [17]. One of the simplest conditional random fields is the chain structure, in this special conditional random field, the chain structure is composed of several character marks. In CRF models with only one order chain, the fully connected subgraph covers the set of the current marker and one marker before it, as well as the maximum connected graph of any subset of the observation sequence. The chained conditional random field is shown in Figure 2, and the set of vertices can be regarded as the maximum connected subgraph.

In the sequence labeling task, random variable *X*={*x*_1_, *x*_2_,…, *x*_*n*_} represents the observable sequence, random variable *Y*={*y*_1_, *y*_2_,…, *y*_*n*_} represents the corresponding marker sequence of the observed sequence [18], and the chained conditional probability distribution of the random variable *Y* is:

(1) $$\begin{array}{c} p\left(y|x\right)=\frac{1}{Z\left(x\right)}\exp\left(\sum_{i,k}\lambda_{k}f_{k}\left(y_{i-1},y_{i},x\right)+\sum_{i,k}\lambda_{k}^{\prime }f_{k}^{\prime }\left(y_{i},x\right)\right). \end{array}$$

In the above formula, *f*_*k*_(*y*_*i*−1_, *y*_*i*_, *x*) is the state feature function for edge and capture mark transfer features. ∑_*i*,*k*_*λ*_*k*_′*f*_*k*_′(*y*_*i*_, *x*) is the non-negative factor for each node. *f*_*k*_′(*y*_*i*_, *x*) is the state feature function that captures the current marked feature for the edge. *λ*_*k*_ and *λ*_*k*_′ are learning model parameters [19], said the weight of characteristic function. *Z*(*x*) is a normalizing factor dependent only on the observation sequence. The specific calculation formula is as follows:

(2) $$\begin{array}{c} Z\left(x\right)=\exp\left(\sum_{i}\sum_{k}\lambda_{k}f_{k}\left(y_{i-1},y_{i},x\right)\right). \end{array}$$

Conditional random field reasoning refers to finding a marker sequence *Y*={*y*_1_, *y*_2_,…, *y*_*n*_} corresponding to the most probable one given an observation sequence *X*={*x*_1_, *x*_2_,…, *x*_*n*_}. In the distribution function of conditional random fields, the normalized factor is completely independent of the marker sequence [20]. Therefore, given the model parameters, the most likely marker sequence can be expressed as:

(3) $$\begin{array}{c} Y^{*}=\arg\max_{y}p\left(y|x\right)=\arg\max_{y}\exp\left(\sum_{i,k}\lambda_{k}f_{k}\left(y_{i-1},y_{i},x\right)+\sum_{i,k}^{k^{\prime }}\lambda_{k}^{\prime }f_{k}^{\prime }\left(y_{i},x\right)\right). \end{array}$$

When the current sequence position is *i* and the current label is *y*, the algorithm can be used to obtain the unnormalized probability value of the optimal label sequence to the current position. Its recursive form is:

(4) $$\begin{array}{c} \theta \left(i,y\right)=\max y^{\prime }\left\{\theta \left(i-1,y^{\prime }*l\sum_{k}\lambda_{k}f_{k}\left(x,y,y^{\prime },i\right)\right)\right\}. \end{array}$$

### 2.2. Key Information Feature Extraction of Digital Archives

Self-organizing feature mapping neural network was proposed by a professor of neural network expert self-organizing feature mapping network of University of Helsinki, Finland in 1981 [21]. This network simulates the function of self-organizing feature mapping of the brain nervous system. It is a kind of competitive learning network, which can carry out self-organizing learning without supervision in learning [22]. This paper uses this method to extract the key information features of different types of digital archives. This can improve the accuracy and efficiency of extracting key information from archives.

**Figure 3:**
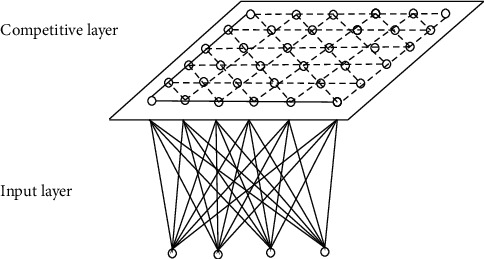
Structure of self-organizing feature mapping neural network.

The structure of self-organizing feature mapping neural network is shown in Figure 3.

We set the number of neurons in the input layer to be *n*, and the number of neurons in the competition layer to be *M*=*m*^2^. The input layer and the competition layer form a two-dimensional planar array. The two layers are connected, and sometimes neurons in the competing layer are also connected by edge inhibition [23]. There are two kinds of connection weights in the network, one is the connection weights of neurons responding to external inputs, and the other is the connection weights between neurons, whose size controls the size of interactions between neurons [24, 25].

**Figure 4:**
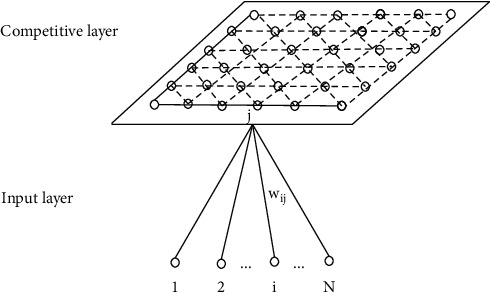
Self-organizing feature mapping network structure after extracting competitive layer neurons.

The connections of neurons at the competitive layer of each input neuron in the self-organizing feature mapping network structure shown in Figure 3 are extracted, as shown in Figure 4.

Set the input mode of the network as *P*_*k*_=(*p*_1_^*k*^, *p*_2_^*k*^,…, *p*_*n*_^*k*^), *k*=1,2,…, *q* and the neuron vector of the competition layer as *A*_*j*_=(*a*_*j*1_, *a*_*j*2_,…, *a*_*jm*_), *j*=1,2,…, *m*. Where *P*_*k*_ is a continuous value and *A*_*j*_ is a numerical quantity. The connection vector between neuron *j* of the competition layer and neuron of the input layer is *W*_*j*_=(*w*_*j*1_, *w*_*j*2_,…, *w*_*jm*_), *j*=1,2,…, *M*.

The self-organizing learning process of the self-organizing feature mapping network can also be described as: for each input of the network, only part of the weight is adjusted to make the weight vector closer to or more deviated from the input vector. This adjustment process is competitive learning. With continuous learning, ownership vectors are separated from each other in vector space, forming a class of patterns representing input space, respectively, which is the clustering function of automatic feature recognition in a self-organizing feature mapping network. The learning and working rules of the network are as follows:

(1) Initialization
Assign the network connection weight {*w*_*ij*_} to the random value *i*=1,2,…, *N*, *j*=1,2,…, *M* in the interval [0, 1]. The initial value of learning rate *η*(*t*), *η*(*t*), 0 < *η*(*t*) < 1 was determined. Determine the initial value *N*_*g*_(0) of neighborhood *N*_*g*_(*t*). Neighborhood *N*_*g*_(*t*) is essentially a region centered on the winning neuron *g* and contains several neurons. This area is generally uniformly symmetrical, most typically a square or circular area. The value of *N*_*g*_(*t*) represents the number of neurons in the neighborhood during the *t*-th learning. Determine the total number of studies *T*.
(2) One of the *q* learning modes *P*_*k*_, *P*_*k*_ is provided to the input layer of the network and normalized. The specific calculation formula is as follows:
  (5) $$\begin{array}{c} {\bar{P}}_{k}=\frac{P_{k}}{P_{k}}=\frac{\left(p_{1}^{k},p_{2}^{k},\ldots ,p_{n}^{k}\right)}{{\left[{\left(p_{1}^{k}\right)}^{2}+{\left(p_{2}^{k}\right)}^{2}+\cdots +{\left(p_{n}^{k}\right)}^{2}\right]}^{1/2}}. \end{array}$$
(3) Normalize the connection weight vector *W*_*j*_=(*w*_*j*1_, *w*_*j*2_,…, *w*_*jN*_) and calculate the Euclidean distance between ${\bar{W}}_{j}$ and ${\bar{P}}_{k}$. The calculation formula of ${\bar{W}}_{j}$ is as follows:
  (6) $$\begin{array}{c} {\bar{W}}_{j}=\frac{W_{k}}{W_{k}}=\frac{\left(w_{1}^{k},w_{2}^{k},\ldots ,w_{n}^{k}\right)}{{\left[{\left(w_{1}^{k}\right)}^{2}+{\left(w_{2}^{k}\right)}^{2}+\cdots +{\left(w_{n}^{k}\right)}^{2}\right]}^{1/2}}. \end{array}$$
The Euclidean distance between ${\bar{W}}_{j}$ and ${\bar{P}}_{k}$ can be calculated by the following formula:
  (7) $$\begin{array}{c} d_{j}=\left[{\sum_{i=1}^{N}\left({\bar{P}}_{i}^{k}-{\bar{W}}_{i}^{k}\right)}^{2}\right],j=1,2,\ldots ,M. \end{array}$$
(4) Find the minimum distance *d*_*g*_ and determine the winning neuron *g*.
  (8) $$\begin{array}{c} d_{g}=\min\left[d_{j}\right]. \end{array}$$
(5) Adjust the connection weights, and modify the connection weights between all neurons in neighborhood *N*_*g*_(*t*) of the competition layer and neurons of the input layer. The specific formula is as follows:
  (9) $$\begin{array}{c} {\bar{w}}_{ji}\left(t+1\right)={\bar{w}}_{ji}\left(t\right)+\eta \left(t\right),\left[{\bar{P}}_{i}^{k}-{\bar{w}}_{ji}\left(t\right)\right]. \end{array}$$
In the above formula, *η*(*t*) is the learning rate at moment *t*.
(6) Select another learning mode to provide to the input layer of the network and return to step (3) until all *q* learning modes are provided to the network.
(7) Updated learning rate *η*(*t*) and neighborhood *N*_*g*_(*t*).
  (10) $$\begin{array}{c} \eta \left(t\right)=\eta \left(0\right)\left(1-\frac{1}{T}\right). \end{array}$$
In the above formula, *η*(0) is the initial learning rate, *t* is the number of learning, and *T* is the total number of learning.
Assume that the coordinate value of a certain neuron *g* in the competition layer in the two-dimensional array is (*x*_*g*_, *y*_*g*_), then the range of neighborhood is point (*x*_*g*_+*N*_*g*_(*t*), *y*_*g*_+*N*_*g*_(*t*)) and point (*x*_*g*_ − *N*_*g*_(*t*), *y*_*g*_ − *N*_*g*_(*t*)) as the square in the upper right corner and the lower left corner, and the modified formula is as follows:
  (11) $$\begin{array}{c} N_{g}\left(t\right)=\text{INT}\left[N_{g}\left(0\right)\left(1-\frac{1}{T}\right)\right]. \end{array}$$
In the above formula, INT(·) is the integral function.
(8) Let *t*=*t*+1, return to step (2), until *t*=*T*.

### 2.3. Key Information Extraction Algorithm of Digital Archives

Key in the process of information extraction, in the digital archives to effectively extract the digital archives of key information, cannot individually understand the individual words of digital archives, and words or similar to each other in the digital archives correlation words combined into a block, a comprehensive understanding of the whole text content and the exact meaning of each word. Therefore, the semantic similarity between words is used as the clustering distance. All the semanemes of a word will form a hierarchical structure similar to a tree according to their upper and lower positional relations, which is traversed through the tree. Finally, the distance between words can be used to judge the similarity of word meaning. The formula for calculating word distance is as follows:

(12) $$\begin{array}{c} \text{Sim}\left(p_{1},p_{2}\right)=\frac{\alpha }{\alpha +\text{dist}\left(p_{1},p_{2}\right)}. \end{array}$$

In the above formula, *p*_1_ and *p*_2_ represent two semesters, which are variable parameters. dist(*p*_1_, *p*_2_) represents the length of the path between two sememes of a word. The semantic origin of describing concepts is divided into four parts: The first basic semantic origin, the symbolic semantic origin, the relational semantic origin, and other independent semantic origin. The overall similarity between concepts is calculated by the following formula:

(13) $$\begin{array}{c} \text{Sim}\left(s_{1},s_{2}\right)=\sum_{i=1}^{n}y_{i}\prod_{j=1}^{i}{\text{Sim}}_{j}\left(p_{1},p_{2}\right). \end{array}$$

In the above formula, *s*_1_ and *s*_2_ represent two concepts, and *y*_*i*_ represents the result of feature extraction. If there are two words *w*_1_ and *w*_2_ in the set, among which word *w*_1_ has *n* concept descriptions and word *w*_2_ has *m* concept descriptions, the maximum similarity between concepts *w*_1_ and *w*_2_ can be used as the semantic similarity of the two words, and the calculation formula is as follows:

(14) $$\begin{array}{c} \text{Sim}\left(w_{1},w_{2}\right)=\underset{i=1,\ldots ,n,j=1,\ldots ,m}{\max}\text{Sim}\left(s_{1i},s_{2j}\right). \end{array}$$

The process of key information extraction algorithm of digital archives is as follows:

Preprocessing: Word segmentation for digital archival text, stop word overconsideration.

**Figure 5:**
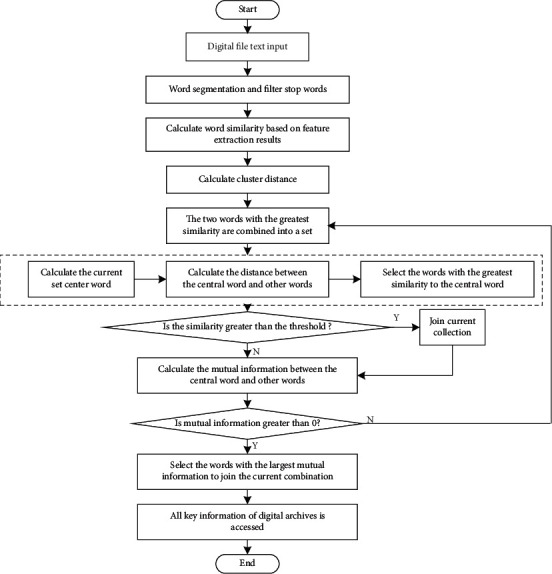
. Key information extraction algorithm flow of different types of digital archives.

Step 1: Calculate all candidate words and semantic similarities between *w*_*i*_ and *w*_*j*_ in digital archival text Sim(*w*_*i*_, *w*_*j*_).
  (1) TF-IDF value is calculated, and word *W*={*W*_1_, *W*_2_,…, *W*_*N*_} with word frequency greater than the threshold *t* is selected as the candidate key information. The calculation formula of TF-IDF value is as follows:
    (15) $$\begin{array}{c} \frac{TF}{IDF_{i}}=\frac{tf_{i}\times \;\log\left(N/n_{i}\right)}{\sqrt{{\sum_{j}\left(tf_{i}\times \;\log\left(N/n_{j}\right)\right)}^{2}}}. \end{array}$$
  In the above formula, *tf*_*i*_ is the number of occurrences of the word in the current digitized archival text, *N* is the total number of digitized archival text, and *n*_*i*_ is the number of digitized archives containing the word *w*_*i*_ in the database.
  (2) During initialization, each word {*W*_*i*_} in the candidate word has a cluster *Z*_*i*_, a total of *n* clusters, and all of them are set with unaccessed markers.
  (3) Among all non-visited word clusters, select the cluster pair (*C*_*l*_, *C*_*k*_) with the largest similarity, that is, the closest distance, by calculating the maximum value of Sim(*w*_*i*_, *w*_*j*_). If Sim(*C*_*l*_, *C*_*k*_) is less than the given threshold, turn to (6); otherwise, merged clusters *C*_*l*_ and *C*_*k*_ are new clusters *C*_0_=*C*_*l*_ ∪ *C*_*k*_. Set to current cluster *C*, *C* to no access flag, *C*_*l*_ and *C*_*k*_ to access flag.
  (4) Calculate the semantic similarity among all unaccessed word clusters, and transfer to (4).
  (5) After clustering, the first *k* words with better quality are selected from each cluster *Z*_*i*_ as the final key information, so as to obtain the candidate word set *W*={*C*_1_, *C*_2_,…, *C*_*m*_}.
Step 2: Treat each word in the text as a set *C*_*i*_, a total of *N*  sets (*N* is the number of words in the text).
Step 3: Select the two sets *C*_*i*_ and *C*_*j*_ with the greatest similarity from the *N* sets, and combine the two sets into a new set *C*.
Step 4: Select the center point of the current set: calculate the mutual information sum of the words in the current set and other words outside the set, and select the word with the largest mutual information value as the center point of the current set. If the calculated mutual information value between words is large, it indicates that they are also relatively large, on the contrary, it indicates that they are relatively small. The mutual information between *w*_*i*_ and *w*_*j*_, that is, the public information between *w*_*i*_ and *w*_*j*_, is calculated as follows:
  (16) $$\begin{array}{c} I\left(w_{i},w_{j}\right)=\log\frac{p\left(w_{i}|w_{j}\right)}{p\left(w_{i}\right)}=\log\frac{p\left(w_{i},w_{j}\right)}{p\left(w_{i}\right)p\left(w_{j}\right)}. \end{array}$$
In the above formula, *p*(*w*_*i*_, *w*_*j*_) is the common frequency of *w*_*i*_ and *w*_*j*_, *p*(*w*_*i*_) is the separate frequency of *w*_*i*_, and *p*(*w*_*j*_) is the separate frequency of *w*_*j*_. According to the above formula, when *I*(*w*_*i*_, *w*_*j*_) > 0, the greater the value, the more public information between *w*_*i*_ and *w*_*j*_ and the stronger the correlation; when *I*(*w*_*i*_, *w*_*j*_)=0, there is less public information between *w*_*i*_ and *w*_*j*_ and the correlation is weak; when *I*(*w*_*i*_, *w*_*j*_) < 0, there is no correlation between *w*_*i*_ and *w*_*j*_.
Step 5: Among other words outside the set, select the word with the highest similarity with the center point of the set. If the similarity value is greater than the threshold, add it to the current set *C*; calculate the mutual information between the central point of the current set and the words outside the set, and add the word with the largest mutual information value to the current set *C*.
Step 6: Turn to step 4 to update the current collection center point until all words are accessed. If the mutual information value between the central point of the set and other words outside the set is less than 0, perform step 3 for the remaining unreachable words until all the words are accessed and divided.
Step 7: In the final cluster set, select its first *K* central words as the key information of the text. The key information extraction algorithm flow of different types of digital archives is shown in Figure 5.

## 3. Results and Discussion

### 3.1. Experimental Scheme

In order to verify the effectiveness of the algorithm designed in this paper to extract archive information, we conducted simulation experiments. This experiment is a simulation experiment, so it is necessary to design the experimental parameters, consider various factors, compare various types of simulation software and computers, and complete the design of environmental parameters of the simulation experiment, as shown in Table 1.

During the experiment, 500 GB digital archives were randomly selected from schools, enterprises, and relevant administrative units as data sets, and 450 GB of them were randomly selected as training sets to train this method. The remaining 50 GB were used as test sets to test the key information extraction performance of different types of digital archives. In order to ensure the objectivity of the experiment, the title and core prompt will be filtered out in the process of extracting key information. Recall rate and accuracy rate are often used as indicators of the key information extraction effect of different types of digital archives. Recall rate *R* and accuracy rate *P* adopted in this experiment are defined as follows:

(17) $$\begin{array}{c} R=\frac{j}{l}\times 100\%. \end{array}$$

In the above formula, *l* represents the number of extracted key information, and *j* represents the actual number of key information.

(18) $$\begin{array}{c} P=\frac{L}{l}\times 100\%. \end{array}$$

In the above formula, *L* represents the amount of key information accurately extracted.

The time-consuming calculation formula for extracting key information of different types of digital archives is as follows:

(19) $$\begin{array}{c} T=\sum_{i=1}^{n}t_{i}. \end{array}$$

In the above formula, *t*_*i*_ represents the time taken for the *i*-th key information extraction step of digital archives.

### 3.2. Analysis and Discussion of Experimental Results

**Figure 6:**
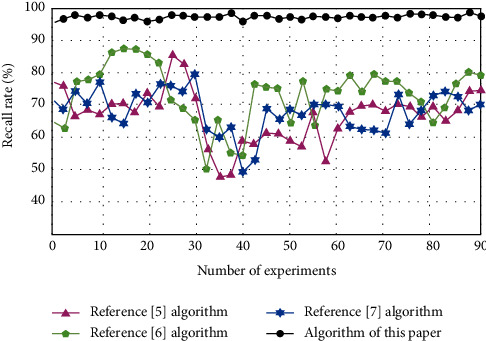
Comparison of recall rate.

The recall rates of key information extraction of different types of digital archives of reference [5] algorithm, reference [6] algorithm, reference [7] algorithm, and algorithm of this paper are compared. The results are shown in Figure 6.

By analyzing the data in Figure 6, we can see that the recall rate of the algorithm in reference [5] changes in the range of 58%–85%, the recall rate of the algorithm in reference [6] changes in the range of 49%–79%, and the recall rate of the algorithm in reference [7] changes in the range of 50%–87%. Compared with the experimental comparison algorithm, the recall rate of the algorithm of this paper changes in the range of 96%–99%, which is always higher than the experimental comparison algorithm, it shows that the key information of digital archives can be extracted comprehensively by using this algorithm, and the integrity is higher.

**Figure 7:**
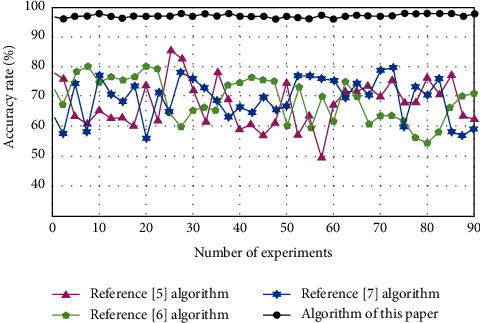
Comparison of accuracy.

The key information extraction accuracy of different types of digital archives of reference [5] algorithm, reference [6] algorithm, reference [7] algorithm, and algorithm of this paper are compared. The results are shown in Figure 7.

By analyzing the data in Figure 7, we can see that the extraction accuracy of key information of digital archives of reference [5] algorithm is 49%–85%, the extraction accuracy of key information of digital archives of reference [6] algorithm is 54%–80%, and the extraction accuracy of key information of digital archives of reference [7] algorithm is 56%–80%. Compared with these algorithms, the extraction accuracy of key information of digital archives of the algorithm of this paper is 96%–98%. On the whole, the key information extraction accuracy of this algorithm is relatively stable, and there is no fluctuation of too high or too low, which indicates that the reliability of this algorithm in extracting key information is high. The accuracy of information extraction is higher, which can achieve the ultimate goal of accurately extracting the key information of different digital archives.

The extraction time of key information of different types of digital archives of reference [5] algorithm, reference [6] algorithm, reference [7] algorithm, and algorithm of this paper are compared. The comparison results are shown in Table 2.

By analyzing the results in Table 2, it can be seen that the average time-consuming of digital archives key information extraction of reference [5] algorithm is 1.41 s, the average time-consuming of digital archives key information extraction of reference [6] algorithm is 1.39 s, and the average time-consuming of digital archives key information extraction of reference [7] algorithm is 1.49 s, which is the highest among the four algorithms. Compared with these algorithms, the average extraction time of key information of digital archives in this algorithm is 0.63 s, which has a shorter extraction time and higher efficiency, and can realize the rapid extraction of key information of digital archives.

To sum up, the recall rate of this algorithm changes in the range of 96%–99%, the accuracy of key information extraction of digital archives is 96%–98%, and the average time-consuming of key information extraction of digital archives is 0.63 s. It can achieve the goal of rapid and accurate extraction of key information of digital archives, solve a variety of problems existing in traditional methods, and can be widely used in many fields.

## 4. Conclusions

With the continuous optimization of cultural operation and management strategies, the level of cultural operation and management has been gradually improved, and digital archives management is an important part of cultural operation and management. Therefore, extracting the key information of different types of digital archives is of great significance to the level of cultural operation and management. Therefore, this paper designs a key information extraction algorithm of different types of digital archives for cultural operation and management. The experimental results show that the recall rate of the algorithm is between 96% and 99%, the accuracy of key information extraction of digital archives is 96%–98%, and the average time-consuming of key information extraction of digital archives is 0.63 s. It can achieve the goal of rapid and accurate extraction of key information of digital archives and can be widely used in cultural operation and management, in order to improve the quality of cultural operation and management to the greatest extent, promote the further development of the cultural industry. However, the convergence of this algorithm is not tested in the process of operation. In order to avoid falling into the local optimum, it is necessary to increase the optimization of the algorithm in future research work to avoid too many iterations or high errors.

## Figures and Tables

**Table 1:** Experimental environment parameters.

Experimental environment parameters	Configuration	Parameter
Hardware environment	CPU	Intel (R)Core (TM)i5-9400
	Frequency	2.90 GHz
	RAM	16.0 GB

Software environment	Operating system	Windows 10
	Analog software language	APDL
	Simulation software	Matlab 7.2

**Table 2:** Extraction time of key information of different types of digital archives.

Number of experiments	Time (s)			
	Reference [5] algorithm	Reference [6] algorithm	Reference [7] algorithm	Algorithm of this paper
10	1.25	1.33	1.47	0.47
20	1.44	1.25	1.58	0.56
30	1.23	0.96	1.56	0.58
40	1.38	1.47	1.47	0.62
50	1.45	1.58	1.52	0.85
60	1.63	1.65	1.35	0.67
70	1.45	1.58	1.47	0.81
80	1.56	1.40	1.62	0.57
90	1.29	1.31	1.41	0.51
Average value	1.41	1.39	1.49	0.63

## Data Availability

The dataset can be accessed upon request.
